# Catalytic Efficiency of Carbon-Cementitious Microfiltration Membrane on the Ozonation-Based Oxidation of Small Molecule Organic Compounds and Its Alkaline Buffering Effect in Aqueous Solution

**DOI:** 10.3390/membranes11080601

**Published:** 2021-08-07

**Authors:** Jingyi Sun, Zhonglin Chen, Shan Liu, Jing Kang, Yuhao Guo, Liming Cai, Jimin Shen, Binyuan Wang, Shengxin Zhao, Zilong Song

**Affiliations:** 1 State Key Laboratory of Urban Water Resource and Environment, School of Environment, Harbin Institute of Technology, Harbin 150090, China; sunjingyihit@163.com (J.S.); zhonglinchen@hit.edu.cn (Z.C.); liumountainhit@163.com (S.L.); guoyuhaohit@163.com (Y.G.); cailiminghit@163.com (L.C.); wangbinyuan2008@163.com (B.W.); shengxin_zhao@163.com (S.Z.); 2 Beijing Key Lab for Source Control Technology of Water Pollution, College of Environmental Science and Engineering, Beijing Forestry University, Beijing 100083, China; tiannonsongzilong@163.com

**Keywords:** carbon-cementitious microfiltration membrane, catalytic ozonation, small molecule organic compounds, alkaline buffering, stability of reuse

## Abstract

In this study, powdered activated carbon (PAC) was added to replace the silica in a cementitious microfiltration membrane (CM) to solve the problems of the low mechanical strength and short lifetime of CMs. The carbon-cementitious microfiltration membrane (CCM) was fabricated by the dry pressing method and cured at room temperature. The bending strength of CCM was 12.69 MPa, which was about three times more than that of CM. The average pore size was 0.129 μm, and was reduced by about 80% compared to that of CM. The addition of PAC did not reduce the degradation efficiency of membrane catalytic ozonation. Because of the strong alkaline buffering ability of CCM, the CCM–ozone coupling process could eliminate the effect of the pH value of the solution. The strong alkaline environment inside the membrane pores effectively accelerated the ozone decomposition and produced oxidizing radicals, which accelerated the reaction rate and improved the utilization rate of ozone. The CCM–catalytic ozonation reaction of organic compounds occurred within the pores and membrane surface, resulting in the pH of the solution belonging to the neutral range. The addition of PAC accelerated the mass transfer and made the pollutants and oxidant react in the membrane pores and on the membrane surface. The reuse experiments of the CCM–ozone coupling process for removing nitrobenzene demonstrated that CCM has good catalytic activity and reuse stability. It broadens the application scope of CCM in the field of drinking water and provides theoretical support for the practical application of CCM.

## 1. Introduction

Cementitious materials are the most widely used construction materials due to their low-costs and easy shaping. Cementitious materials such as alkali-activated materials (AAMs) are also used in water treatment [1] due to their good physicochemical stability [2], porous structure [3], and high mechanical strength [4]. Ge et al. [5] made AAMs into a sinter-free, self-supporting inorganic membrane for the adsorption of Ni^2+^ in solution with a capacity of 43.36 mg/g. Cilla et al. [6] added vegetable and animal oils to a porous geopolymer suspension to generate surfactants in situ. Asim et al. [7] used AAMs as catalysts for the removal of organic pollution in water and the air by adjusting the surface structure, chemical properties, and porosity of AAMs. Novais et al. [8,9] found that AAMs in solution overflowed OH^−^ continuously, leading to an increase in solution pH with increasing time, which eventually stabilized around 10, demonstrating their potential as pH regulators in applications requiring a high buffering capacity.

Membrane separation is widely used in the water treatment process because it is environmentally friendly and does not produce by-products pollution, while a high-pressure membrane with smaller pore size and stronger separation ability needs a higher driving force to run and consumes more energy. The separation ability of a low-pressure membrane with a larger pore size cannot reject small molecular organic compounds. In recent years, membranes with catalytic properties to compensate for the overly large pore size of the low-pressure membrane have gradually become a hot spot. In these studies, there are few studies on membrane catalytic ozonation to degrade organic pollutants. Cheng et al. [10] incorporated Mn oxide and ceramic membranes to catalytic ozonation to control membrane fouling and improved the degradation rate of *p*-chloronitrobenzene (*p*-CNB) by 19.4%. Guo et al. [11,12] fabricated a catalytic ceramic membrane with CuMn_2_O_4_ to degrade UV absorber benzophenone-3 by catalytic ozone-base oxidation, and increased the removal rate by 27.4%. Zhang et al. [13] prepared membrane-confined iron oxychloride nanocatalysts for catalytic heterogeneous Fenton. The reaction rate constant of *p*-chlorobenzoic acid (*p*-CBA) is 0.223 s^−1^, while the membrane fabrication process of these studies is relatively complicated, or the base membrane needs a high sintering temperature which may increase fabrication costs. There are few studies conducted to fabricate a membrane using cementitious material itself for the catalytic ozonation of organic pollutants. Our research team [14] fabricated a microfiltration membrane (MF) by using quartz and cement for catalytic ozonation of *p*-CNB in water with a 50% higher reaction rate than ozone oxidation alone. Furthermore, the prepared silicate-based membrane was used to catalyze the ozone-based oxidation of iopamidol in aqueous solution [15]. The reaction ratio constant of iopamidol removal by catalytic ozonation is 0.2866 min^−1^. The catalytic ozonation pathway and the generated intermediates were analyzed. In the previous study [16], a low-cost aluminosilicate-based microfiltration membrane was obtained by dry pressing with cementitious materials and silica powder as the main raw materials. For the catalytic ozonation of benzophenone-4 (BP-4), the reaction rate *k*_obs_ was 0.31 min^−1^ which was nearly three times higher than that of ozone alone, and the mineralization increased by 20%. However, in practical application, the mechanical strength of a silicate-based membrane is generally low, about 4–5 MPa, and it is prone to fracture during long-term use, which affects its service lifetime.

Carbon materials are often added to cement as additives to improve the stability and mechanical strength of cement [17,18]. The addition of carbon materials does not affect the type of cement hydration products [19], and carbon materials act as substrates to promote the generation of C-S-H and accelerate hydration. In addition, the surface of carbon materials is rich in a variety of functional groups and they have their own pore structure, which lead them to be the most commonly used adsorbents for water treatment [20]. It is reported that activated carbon materials can also catalyze the generation of oxidizing radicals in ozonation and improve the utilization efficiency of ozone, thereby increasing the removal rate of organic matter [21,22,23,24] without the problem of secondary leakage from metal oxide catalysts, and eliminated the need for separate activated carbon regeneration. Powdered activated carbon (PAC) as an additive added to cementitious membranes to improve the mechanical strength and broaden the range of applications has not been reported.

In this study, a MF with high mechanical properties and a small average pore size was fabricated by PAC and cementitious materials. The removal efficiency and reaction kinetics of organic compounds with different functional groups by ozonation catalyzed by the carbon-cementitious microfiltration membrane (CCM) were investigated. The alkaline buffering effect of the membrane has a leveling effect on the impact of solution pH on the oxidation system. The changing trends for the solution pH in the presence and absence of organics were investigated. The accelerating effect of membrane pore size on the removal of organic matter was also explored. It was demonstrated that CCM could improve the utilization of ozone. The stability of membrane catalytic ozone performance and the feasibility of long-term use were verified by reuse experiments of the CCM–ozone coupling process. This research could provide support for the practical application of CCM.

## 2. Materials and Methods

### 2.1. Materials and Reagents

PAC (Tianjin Kermel Chemical Co., Ltd., Tianjin, China) and cementitious powder (PO 42.5, Tian E^®^, Harbin, China) were the main raw materials for the membrane fabrication. SiO_2_ partials (99.5%, 500 nm) was used as the model macromolecular to evaluate the retention of the membranes, which was purchased from Macklin (Shanghai, China). Six model compounds with different p*K*_a_ were chosen to verify the universality of CCM–catalyzed ozonation. Nitrobenzene (99% purity), *p*-CNB (99% purity), *p*-chloroaniline (*p*-CA) (98% purity), *p*-chlorophenol (*p*-CP) (99% purity), and *p*-CBA (99% purity) were all purchased from Sigma-Aldrich (Sigma-Aldrich Inc., St. Louis, MI, USA). BP-4 (98% purity) was purchased from J&K (Beijing, China). These organic pollutants were all configured to 100 mg/L as stock solutions. Na_2_SO_3_ was used as the quencher of the catalytic ozonation, which was obtained by Benchmark (Tianjin, China) and stocked at a concentration of 0.1 mol/L. The saturated KI solution was prepared as the ozone exhaust gas absorbent and purchased from Sinopharm Chemical Reagent Co., Ltd. (Shanghai, China). Indigo Carmine was obtained from Shanghai Experiment Reagent Co., Ltd. (Shanghai, China). Na_2_S_2_O_3_ was purchased from Tianjin Baishi Chemical Industry Co., Ltd. (Tianjin, China). Ultrapure water used in all experiments was supplied by the Milli-Q^®^ system.

### 2.2. Characterization of CCM

CCM was fabricated with cementitious materials by doping 10 wt% PAC, adding deionized water to drier powder at water-to-cementitious = 0.2, via the dry pressed molding method. The wet paste was put into a mold and pressed at a constant pressure of 6 MPa for 1 min, and then put into a standard curing box (Shanghai Bluepard Instruments Co. Ltd., Shanghai, China) at 20 °C and 90% relative humidity (RH) curing for 1 d. The paste then continued to cure for 13 d after demolding. The diameter of the CCM was 50 mm and the thickness was 5 mm.

The porosity of the membranes was measured based on the standard method specified by ASTM C20-00: Archimedes’ method. The average pore size of the CCM was measured by mercury intrusion porosimeter (MIP, Micromertics AutoPore IV 9500 V1.09, Norcross, GA, USA). The bending strength was used to express the mechanical strength of the membrane, as tested by a universal strength testing machine (Instron 5569, Instron Corp., Norfolk County, MA, USA). The pure water flux (PWF) of the membrane was measured to investigate the filtration performance of the membrane by measuring the volume of pure water filtered through the membrane in unit time. The rejection rate of SiO_2_ by the membrane was expressed by the change in turbidity of the influent and the effluent passing through the membrane. The calculation of the rejection rate is shown in Text S1.

### 2.3. CCM Catalytic Ozonation Process

**Figure 1:**
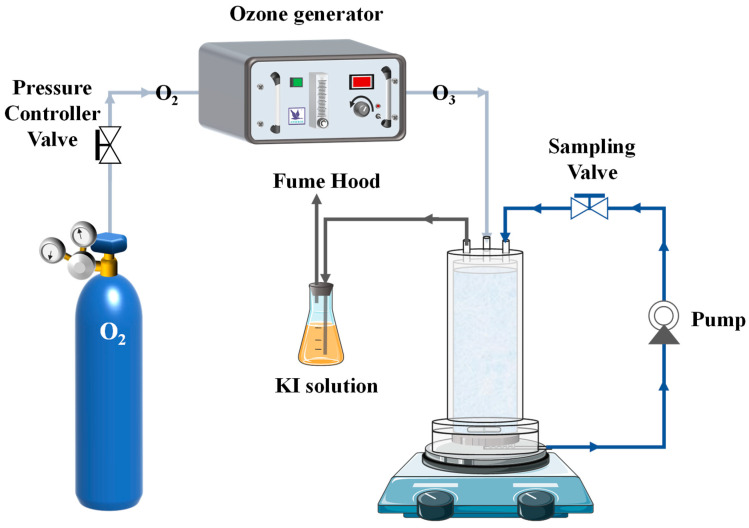
Schematic diagram of membrane-catalyzed ozonation for organic pollutants removal.

In order to investigate the water permeability and catalytic effect on the ozone of the membrane, a semi-batch membrane-catalytic ozonation experiment for the removal of organic pollutants reactor was designed, and the schematic diagram is shown in Figure 1. A 250 mL feed water with 0.064 mM organic concentration was prepared in a closed reactor, and the organic solution was continuously circulated through the membrane by the power of a peristaltic pump (BT 100-2J, Longer Precision Pump Co., Ltd., Baoding, China). At the same time, ozone gas was continuously added into the reactor and the flow rate was adjusted to maintain the ozone concentration in the solution at 0.5 mg/L. Ozone was generated from dry high purity oxygen through an ozone generator (COM-AD-01 Anseros, Tübingen-Hirschau, Germany). The residual ozone gas was absorbed by saturated KI solution. As the reaction proceeded, samples were taken rapidly at certain times and the reaction was quenched by adding 0.1 mol/L Na_2_SO_3_ solution to the samples. The concentration of each organic pollutant was measured at the corresponding time point.

To investigate the effect of the membrane alkaline buffering on the pH in solution, the reactor in Figure 1 was replaced by a three-neck flask. One of the necks was continuously injecting ozone gas, the second neck was put in a pH meter probe, and the third neck was used for sampling or sealing. The schematic diagram is shown in Figure S1. Putting the CCM alone into the pure water was named the membrane alone system, continuously injecting ozone gas into the solution was named the Ozone/CCM system, and the solution with the organic pollutant added was named the Ozone/CCM + OR system. The pH value was determined using a pHS-3C meter (Beijing Kewei Yongxing Instrument CO., LTD., Beijing, China). The pH value of the membrane surface was measured by a special pH indicator paper (test range 8.2–10.0, SSS reagent, Shanghai, China), the detection precision can be to one decimal place. After the membrane catalytic ozone-based oxidation experiments the membrane was taken out, and the surface moisture was wiped away. We put the special indicator paper on the surface of the membrane to test the pH of the membrane surface.

### 2.4. Analytical Methods

The determination of the organic compounds was carried out by ultra-performance liquid chromatography (UPLC; Agilent 1290 Infinity II, Santa Clara, CA, USA). The determination conditions of six organic compounds are shown in Text S2.

The ozone concentration in the solution was determined by the indigo method at λ = 610 nm by UV-Vis spectrophotometry (T6 Persee, Beijing, China).

The determination of gaseous ozone was analyzed by the iodometric method. The principle is that the strong oxidant ozone reacts with potassium iodide aqueous solution to generate free iodine, ozone is reduced to oxygen, free iodine is colored and then titrated using sodium thiosulfate standard solution, the free iodine becomes sodium iodide, and the end point of the reaction is the complete discoloration of the solution. The reaction equation is as Equations (1) and (2):

(1) $$\mathrm{KI}+O_{3}+H_{2}O\to I_{2}+O_{2}+2\mathrm{KOH}$$

(2) $${2\mathrm{Na}}_{2}S_{2}O_{3}+I_{2}\to {\mathrm{Na}}_{2}S_{4}O_{6}+2\mathrm{NaI}$$

0.1000 mo1/L Na_2_S_2_O_3_ standard solution concentration and 20% KI solution were prepared. We measured out 20 mL of potassium iodide solution, then added 350 mL of distilled water. When the ozone generator ran steadily a sample was taken at the outlet gas of the ozone generator, which was passed into the absorption bottle to absorb the ozone and to measure the amount of gas passage with a gas flow meter to 1000 mL. 5 mL of (1 + 5) sulfuric acid solution was added immediately after stopping collection of the sample and was shaken well, then left to stand for 5 min. Then the mixture was titrated with 0.1000 mol/L sodium thiosulfate standard solution, a few drops of starch solution were added when the solution was light yellow, and they were titrated carefully and rapidly until the color disappeared. The volume of sodium thiosulfate standard solution was recorded. The concentration of gaseous ozone was calculated as in Equation (3).

(3) $$m_{\left(O_{3}\right)}=C_{{\mathrm{Na}}_{2}S_{2}O_{3}}\times V_{{\mathrm{Na}}_{2}S_{2}O_{3}}\times \frac{24000}{V_{O_{3}}}$$

where m_(O3)_ is the mass concentration of O_3_ (mg/L), C_(Na2S2O3)_ is the concentration of Na_2_S_2_O_3_ standard solution as 0.1000 mol/L, V_(Na2S2O3)_ is the volume of Na_2_S_2_O_3_ standard solution titrated, and V_(O3)_ is the volume of ozone gas passed as 1000 mL.

The ozone utilization efficiency was calculated as in Equation (4) [25].

(4) $$U\;\left(\%\right)=\frac{{\left[O_{3}\right]}_{I}-{\left[O_{3}\right]}_{O}-{\left[O_{3}\right]}_{\mathrm{aq}}}{{\left[O_{3}\right]}_{I}}$$

where U is the ozone utilization rate (%), [O_3_]_I_ is the ozone input over time at the reactor inlet (mg), [O_3_]_O_ is ozone content over time at the reactor outlet (mg), and [O_3_]_aq_ is the dissolved ozone content in the reactor (mg).

## 3. Results and Discussion

### 3.1. Characterization of CCM

**Figure 2:**
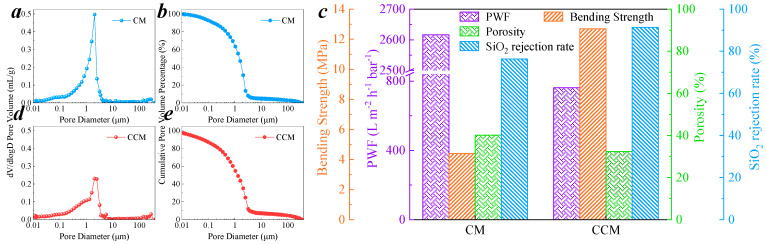
Performance comparison between CMs and CCMs. (**a**) Pore size distribution; (**b**) cumulative pore volume of CM, (**c**) Bending strength; PWF; porosity and SiO_2_ rejection rate of CM and CCM, (**d**) Pore size distribution; (**e**) cumulative pore volume of CCM. Concentrations: Initial turbidity of SiO_2_ is 20 NTU; TMP = 20 kPa. Membrane fabrication condition: molding pressure is 6 MPa, w/c = 0.2, 20 °C, curing for 14 days in 90% RH.

In the previous study [16], an aluminosilicate-based microfiltration membrane (CM) was prepared by silica powder and cementitious materials. The pore size distribution of CM is shown in Figure 2a, the average pore size is 0.633 μm. The cumulative pore volume percentage of CM was calculated, as shown in Figure 2b, the volume ratio of pore size in MF range (0.1–10 μm) [26] was 81.88%. The membrane porosity is 37.9%, PWF is 2605.78 L/m^2^/h/bar, and the membrane bending strength is 4.41 MPa. In the practical use process, it was found that the strength of CM could not support the membrane well for multiple reuses and it was prone to membrane fragmentation, which affected the long-term stability of the membrane.

It has been reported that adding a certain amount of carbon material into the cementitious materials can improve the stability and mechanical strength of the materials [27,28]. Therefore, PAC was used as a substitute for silicon powder to prepare CCM. The pore size distribution of the membrane was measured and shown in Figure 2d, the average pore size of the CCM was 0.129 μm, which was reduced by about 80% compared to that of CM. As shown in Figure 2e, the volume ratio of pore size in MF range was 78.79%, which is similar to the pore size range of CM. SiO_2_ particle with 500 nm diameter was chosen to compare the rejection property of CM and CCM, the diameter of which is between the average pore size of CM and CCM. The SiO_2_ rejection rate by CM and CCM was 76.33% and 91.34%, respectively, at trans-membrane pressure (TMP) = 20 kPa, as shown in Figure 2c. This result proved the pore size of CCM was smaller than that of CM. Although the diameter of SiO_2_ particle was smaller than the average pore size of CCM, the rejection rate still did not reach 100%. This result was corroborated by the pore size distribution of the membrane, which showed that there were still about 7% coarse pores (>10 μm) in the membrane [26]. The membrane porosity was 32.4%, PWF was 762.11 L/m^2^/h/bar, and bending strength was 12.69 MPa. Compared with CM, the bending strength increased by three times. Although the CCM has a certain loss of PWF compared to that of CM, it still has a large PWF compared to other inorganic membranes [28,29,30]. These properties greatly ensure the stability and reusability of CCM during use.

In addition, when using BP-4 as a model compound, the degradation of organic compounds by catalytic ozonation was studied. It was found that in the intermittent ozonation process, ozone was quickly decomposed and there was a rapid removal of BP-4 only in the initial stage of the reaction. With the reaction time, the CM–ozone coupling process and ozone alone had no effect on the removal of BP-4, while the removal rate of BP-4 by the CCM–ozone coupling process was still increasing, as shown in Figure S2. This was due to the adsorption of PAC doped in CCM on BP-4, which further broadens the application of cementitious membranes.

### 3.2. Degradation of Organic Pollutants by Membrane-Catalyzed Ozone

**Figure 3:**
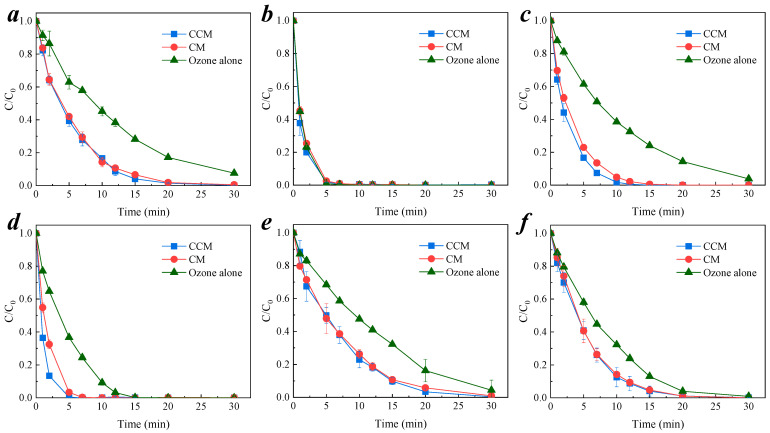
Degradation of organic pollutants by CM– and CCM–catalyzed ozonation. (**a**) nitrobenzene; (**b**) *p*-CA; (**c**) BP-4; (**d**) *p*-CP; (**e**) *p*-CNB; (**f**) *p*-CBA. Conditions: pH = 6.9 ± 0.1, [O_3_] = 0.5 mg/L, [nitrobenzene]_0_ = [*p*-CA]_0_ = [BP-4]_0_ = [*p*-CP]_0_ = [*p*-CNB]_0_ = [*p*-CBA]_0_ = 0.064 mM.

To verify the broad spectrum of CCM–catalyzed ozonation of organic compounds, six small molecule organic compounds with different functional groups—nitrobenzene, *p*-CA, BP-4, *p*-CP, *p*-CNB, and *p*-CBA—were selected as model compounds. Under neutral conditions of pH = 6.9 ± 0.1, ozone gas was continuously added into the reaction. The concentration of ozone in the solution was 0.5 mg/L and the concentration of organic feed water was 0.064 mM. The degradation of six model compounds by membrane-catalyzed ozone is shown in Figure 3a–f. It can be seen that the removal efficiency of the other organic pollutants was significantly improved except for *p*-CA, which is easily oxidized by ozone molecules [31].

In addition, the efficiency of ozone-catalyzed oxidation of organic compounds did not change significantly, although silica was replaced by PAC doped into cementitious materials. The kinetic fitting of the six compounds removal rates is shown in Figure S3a–f. The reactions of both membrane-catalyzed ozone-based oxidation and sole ozone oxidation for the removal of organic compounds were consistent with the pseudo-first-order kinetics. The calculated reaction kinetic constant *k*_obs_ is shown in Table 1; the *k*_obs_ values of the reactions of the CCM-catalyzed ozone-based process were 1.6–4.0 times higher than the *k*_obs_ value of the sole ozone process. Compared with the *k*_obs_ of the CM–catalytic ozonation process, the *k*_obs_ values of the CCM-catalyzed ozone were slightly improved. Wang et al. [32] used an electro-grounded active carbon–ozone process to degrade nitrobenzene, the concentration of nitrobenzene is 6.00 mg/L and the ozone concentration is 2.3 mg/L, the reaction kinetic constants *k*_obs_ is 0.078 min^−1^. Chen et al. [33] studied the efficient degradation of nitrobenzene by membrane separation system with MgO (111), and the *k*_obs_ is 0.07 min^−1^. The concentration of ozone and nitrobenzene is 5.0 mg/L and 50.0 mg/L, respectively. Song et al. [34] used r-GO-ceramic-ultrafiltration membrane catalytic ozonation to remove *p*-CBA, the *k*_obs_ is 0.279 min^−1^, while the concentration of ozone is 4 times higher than our studies. Compared with the reported studies, CCM showed a good catalytic performance. This result demonstrates the feasibility of using PAC as an additive instead of silica powder for membrane catalytic ozone capacity.

The normalized water permeance (*J*/*J*_0_) of CCM remained unchanged after being used in the catalytic ozonation experiment as shown in Figure S4. This indicated the parent small organic compounds and the degradation products did not block the pore. In addition, some studies reported that the catalytic membrane has the self-cleaning property [25]. This indicated CCM also has this self-cleaning property.

### 3.3. Effect of pH on Membrane Catalytic Ozonation

**Figure 4:**
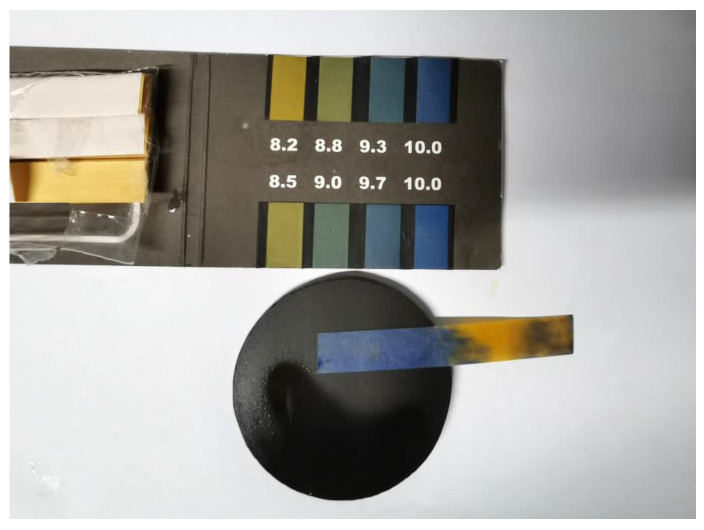
Surface pH of CCM in the process of CCM–catalyzed ozonation for organic pollutants removal.

The pH in solution is an important indicator of the rate of an ozone-based oxidation reaction [35]. When experiments on the effect of pH on the removal of organic pollutants by CCM-catalyzed ozone were carried out with a concentration of 0.064 mM nitrobenzene as the model, it was found that the final pH of the solution was in the neutral range. However, when tested with special pH indicator paper, the membrane surface was strongly alkaline with a pH of about 10, as shown in Figure 4. To explore this phenomenon, this section monitors the change trends of pH in the solution with the membrane alone system, the Ozone/CCM system, and the Ozone/CCM + OR system, with initial pH values of 4.0, 5.0, 6.0, 7.0, 8.0, 9.0, and 10.0.

#### 3.3.1. Change Trend of pH in the Degradation Process

**Figure 5:**
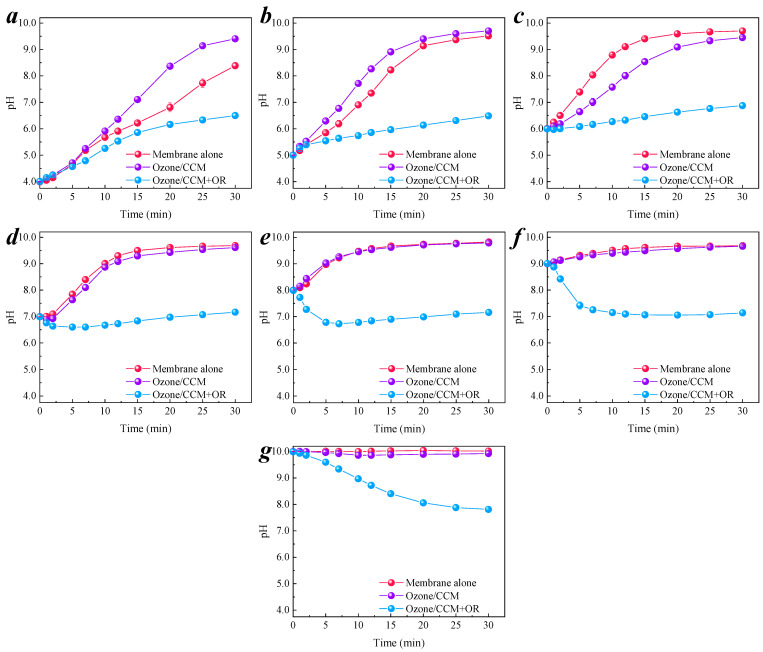
Effect of initial pH on pH change trend in different reaction systems. (**a**) pH_initial_ = 4.0; (**b**) pH_initial_ = 5.0; (**c**) pH_initial_ = 6.0; (**d**) pH_initial_ = 7.0; (**e**) pH_initial_ = 8.0; (**f**) pH_initial_ = 9.0; (**g**) pH_initial_ = 10.0. Conditions: [O_3_] = 0.5 mg/L, [nitrobenzene]_0_ = 0.064 mM.

**Figure 6:**
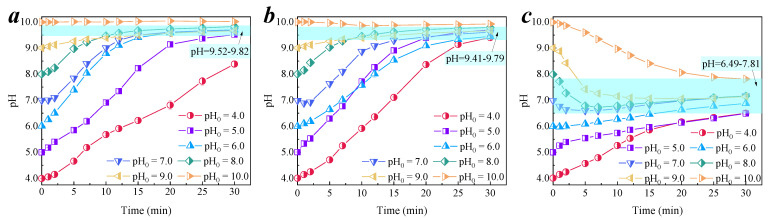
Change trends of pH in different reaction systems. (**a**) Membrane alone; (**b**) Ozone/CCM system; (**c**) Ozone/CCM + OR system. Conditions: [O_3_] = 0.5 mg/L, [nitrobenzene]_0_ = 0.064 mM.

The pH trends of the three systems at each initial pH condition are shown in Figure 5a–g. It can be observed that the pH of both the membrane alone and the Ozone/CCM system increased with time. The change trend of pH increased for the membrane alone system and was slightly faster than that of the Ozone/CCM system. This is because the addition of ozone reacts with OH^−^ in the solution and consumes a certain amount of OH^−^ [36], resulting in a slightly slower pH increase. However, after the addition of the organic pollutant, the pH increased with time when the initial pH < 7.0, (i.e., the initial solution was acidic), and decreased with time when the initial pH > 7.0, (i.e., the initial solution was alkaline). The pH trends of the three systems are shown in Figure 6a–c. It can be seen that the final pH of the membrane alone was slightly larger than that of the Ozone/CCM system, and the pH values were 9.52–9.82 and 9.41–9.79, respectively. This is due to the strong alkaline buffering effect of the cementitious material [9], which has a leveling effect on the initial pH of the solution, avoiding the effect of the solution pH itself. The final pH of the CCM was less than the reported final stable pH of 10.5 for the cementitious material. This may be due to the large number of oxygen-containing functional groups on the surface of the PAC doped in the CCM, which can neutralize the OH^−^ in the solution, resulting in the final pH of the CCM solution being around 9.6.

When organic compounds were added to the system, the final pH of the Ozone/CCM + OR system was 6.49–7.81, which was in the neutral range and met the EPA drinking water effluent quality standard. This may be due to the good electron transfer properties of carbon materials [37], which can accelerate the mass transfer between the oxidized substances generated inside the membrane pores and the organic substances inside the membrane pores so that the OH^−^ in the solution is rapidly consumed, the neutralization of OH^−^ is accelerated, and the solution is neutral. The alkalinity of the membrane surface indicates that the CCM-catalyzed ozonation of organics occurred within the membrane pores and on the membrane surface. This result can provide experimental support for the application of CCM in deep drinking water treatment.

#### 3.3.2. Transmembrane Removal of Organic Compound

**Figure 7:**
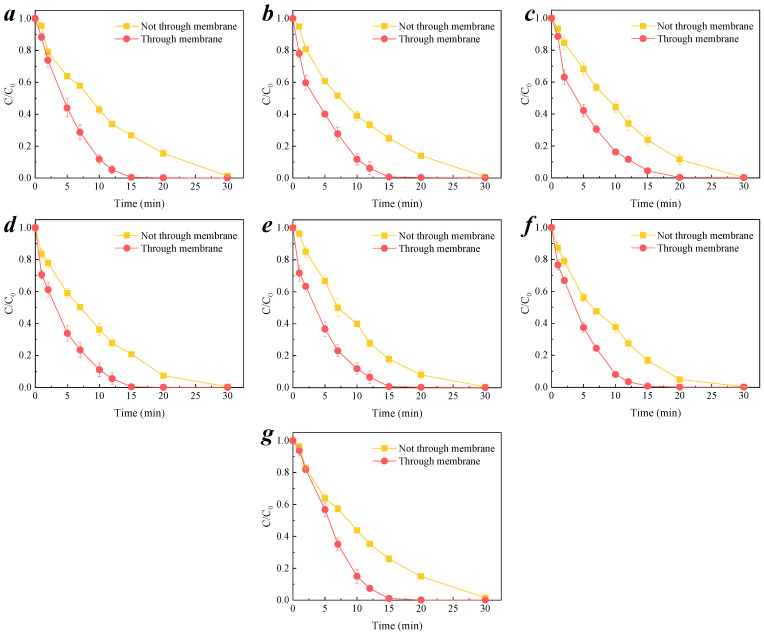
Degradation of nitrobenzene by CCM–catalyzed ozonation with solutions through or not through the membranes in different initial pH conditions. (**a**) pH_initial_ = 4.0; (**b**) pH_initial_ = 5.0; (**c**) pH_initial_ = 6.0; (**d**) pH_initial_ = 7.0; (**e**) pH_initial_ = 8.0; (**f**) pH_initial_ = 9.0; (**g**) pH_initial_ = 10.0. Conditions: [O_3_] = 0.5 mg/L; [nitrobenzene]_0_ = 0.064 mM.

To investigate the effect of solution pH on the alkaline environment inside the membrane pores, the membrane was put into the nitrobenzene solution and not in the through-membrane system. The experiment set-up was the same as pH value measured, as shown in Figure S1. The solution was continuously stirred by a magnetic stirrer, and the initial concentration of nitrobenzene is 0.064 mM. The solution was not filtered through the membrane, and the membrane was just like an alkaline buffering. This was compared with the feed water filter through the CCM system, for which the experimental set-up schematic is shown in Figure 1. The degradation efficiency of nitrobenzene in both systems is shown in Figure 7a–g. It can be observed that the degradation efficiency of the system with feed water continuously filtering through the membrane process was obviously faster than that of the system with the solution not filtering through membrane, regardless of the initial pH. The kinetic fits of nitrobenzene by CCM-catalyzed ozonation for the through-membrane and not through-membrane reaction systems at the seven initial pHs were calculated, and the *k*_obs_ values are listed in Table S1. The *k*_obs_ for the solution of the through-membrane system was about twice as large as that for the not through-membrane system. The micron-sized membrane pores act as a concentrator of organic compound as it passes through the membrane pores. The strong alkaline environment within the membrane pores enhanced the activation efficiency of ozone, generating high concentrations of ·OH, which are more likely to attack organic molecules when close proximity to the contaminants [25,38]. Overall, the reaction rate (*k*_obs_) of the through-membrane system was higher than that of the not through-membrane.

**Figure 8:**
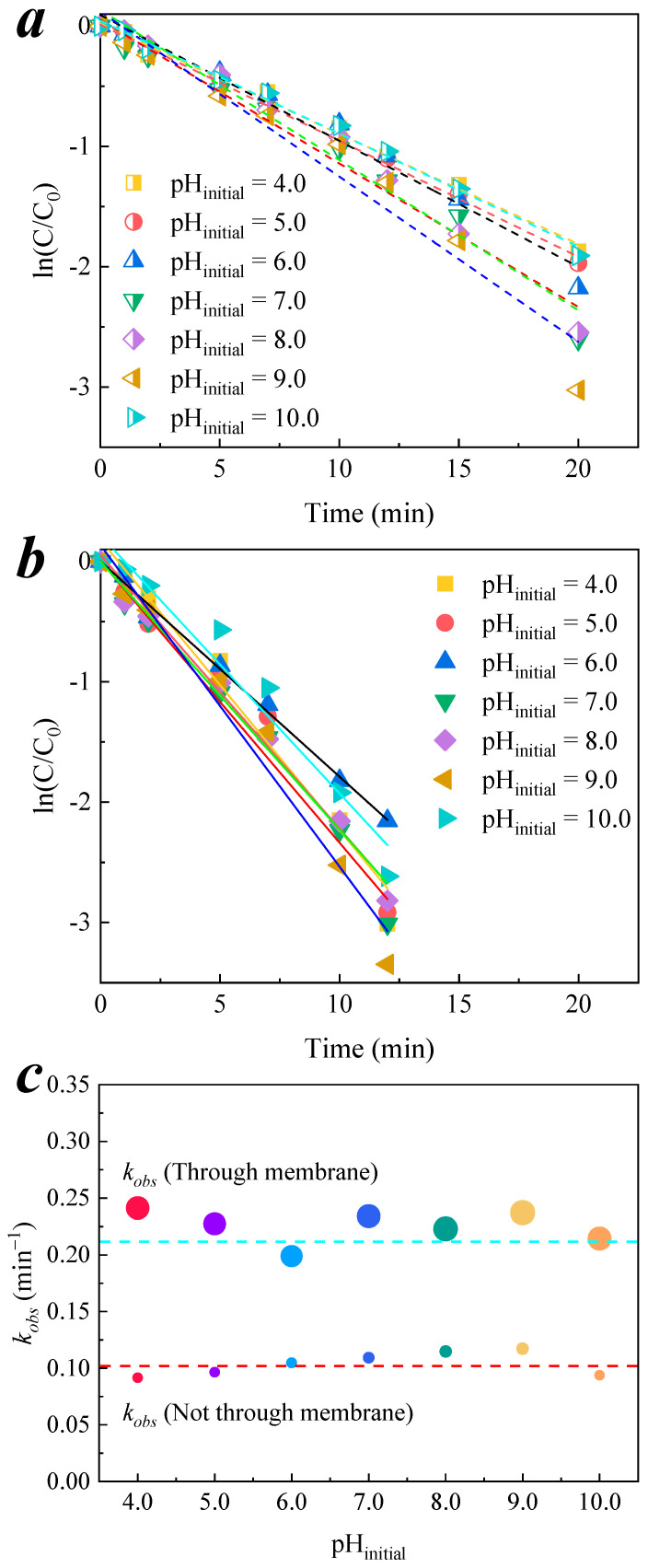
Reaction kinetics of nitrobenzene by CCM–catalyzed ozonation in different initial pH systems. (**a**) Pseudo-first-order kinetic fitting not through membrane process; (**b**) fitting through membrane process; (**c**) *k*_obs_ of through and not through membrane process. Conditions: pH = 6.9 ± 0.1, [O_3_] = 0.5 mg/L, [nitrobenzene]_0_ = 0.064 mM.

For the contaminant, no matter whether in a through or not through CCM system, the seven initial pHs had less of an effect on the reaction rate constants; their pseudo-first-order kinetic fitting is shown in Figure 8a,b. Comparing the *k*_obs_ with different initial pH values for the two systems, as shown in Figure 8c, it can be seen that the *k*_obs_ values with an alkaline initial pH were slightly higher than that with an acidic initial pH. The standard deviations of the seven initial pHs *k*_obs_ values were calculated to be 0.010 and 0.014 for the not through- and through-CCMs, respectively, and the coefficient of variations were 0.099 and 0.065, respectively. The values are extremely low, indicating that the dispersion of *k*_obs_ in the seven initial pHs was very low. It proves that the alkaline buffering effect of CCM is strong and has a leveling effect on the initial pH of the solution.

#### 3.3.3. Decomposition and Utilization of Ozone

Previous studies have shown that [16] the cementitious membrane has a large amount of alkaline calcium silicate hydrate (C-S-H) and Ca(OH)_2_ inside the membrane pores [39], which significantly increases the pH of the solution inside the membrane pores, as shown in Figure 6a. When the dissolved ozone solution flows through the membrane pores, the alkaline environment inside the pores promotes the decomposition of ozone and generates more hydroxyl radicals with a strong oxidizing ability (∙OH) by the following chain reactions in Equations (5)–(8) [32].

(5) $$O_{3}+{\mathrm{OH}}^{-}\to {\mathrm{HO}}_{2}^{-}+O_{2}$$

(6) $$O_{3}{+\mathrm{HO}}_{2}^{-}\to O_{3}^{∙-}{+\mathrm{HO}}_{2}^{-}$$

(7) $$O_{3}^{∙-}{+H}^{+}↔{\mathrm{HO}}_{3}^{∙}$$

(8) $${\mathrm{HO}}_{3}^{∙}\to ∙{\mathrm{OH}+O}_{2}$$

**Figure 9:**
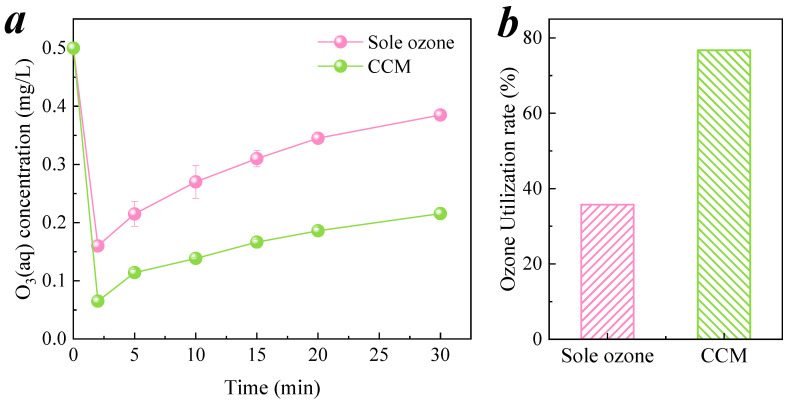
(**a**) Ozone decomposition and (**b**) ozone utilization rate in the process of CCM–catalyzed ozonation of nitrobenzene. Conditions: pH = 6.9 ± 0.1, [O_3_]_aq_ = 0.5 mg/L, [nitrobenzene]_0_ = 0.064 mM, ozone gas flow velocity = 0.3 L/min.

At the same time, the hydration products of cementitious materials are calcium silicate, aluminosilicate, and calcium alumina containing Al-O and Fe-O bonds [40], and these oxides have been proven to catalyze ozone decomposition to produce free radicals [41,42]. Therefore, the decomposition rate of ozone in the presence of CCM was higher than that of sole ozone, as shown in Figure 9a. When pollutants were added, the free radicals rapidly oxidized and degraded the pollutants, which made the free radicals produced in the ozone decomposition reaction move to the right and further promote the ozone decomposition. The ozone utilization rates of sole ozone and membrane catalyzed ozonation were 35.72% and 76.72%, respectively, as shown in Figure 9b. According to Equation (4), membrane catalysis can effectively improve the utilization rate of ozone.

### 3.4. Reusability of CCM

**Figure 10:**
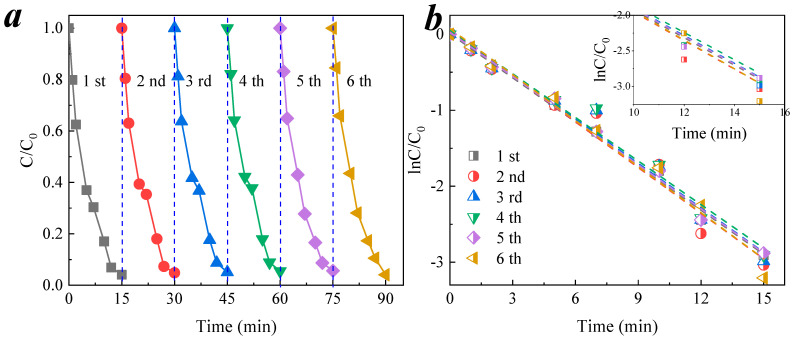
Reuse of CCM–ozone catalysis process in degradation of nitrobenzene. (**a**) Degradation of nitrobenzene by CCM–catalyzed ozonation; (**b**) Reaction kinetics of nitrobenzene by CCM–catalyzed ozonation. Conditions: pH = 6.9 ± 0.1, [O_3_] = 0.5 mg/L, [nitrobenzene]_0_ = 0.064 mM.

The membrane reusability is also an important indicator for characterizing the application of the membrane in a practical treatment process. Using nitrobenzene as the model compound, the CCM–ozone coupling process for nitrobenzene removal was repeated six times under the same conditions, and the obtained nitrobenzene removal rate is shown in Figure 10a. It can be seen that the degradation rate did not change significantly in the six times it was tested. The pseudo-first-order kinetic fit of the results, as shown in Figure 10b, indicates that the *k*_obs_ of the six reuses fluctuated slightly from 0.193 min^−1^ to 0.202 min^−1^.

**Figure 11:**
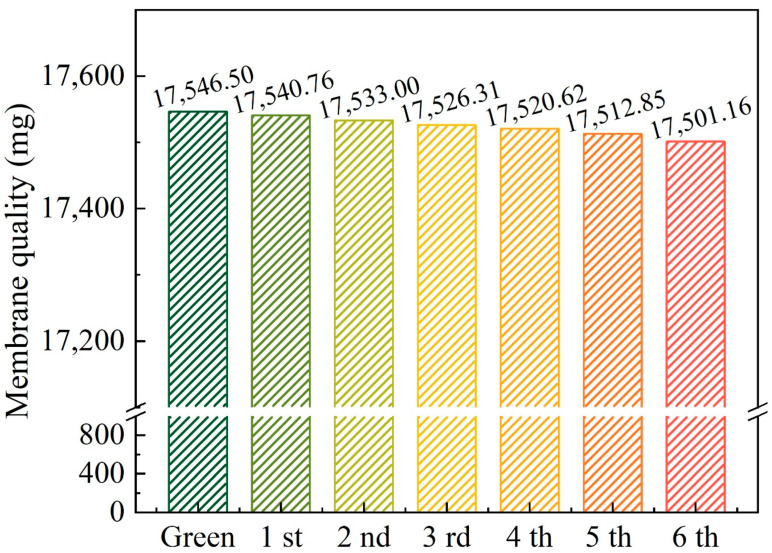
Quality loss of CCMs in six reuse experiments. Conditions: pH = 6.9 ± 0.1, [O_3_] = 0.5 mg/L, [BP-4]_0_ = 0.064 mM.

To investigate the effect of ozone on the catalytic active substances on the membrane surface, the dried membrane after each experiment was weighed. The mass loss of the membrane is shown in Figure 11, and the quality loss rate after six reuses was 0.25%, which is very low. This demonstrates that CCM catalyzes ozone in a stable and continuous manner without causing excessive loss to the membrane. These results are of guiding significance for the practical application of CCM in the water treatment process.

## 4. Conclusions

In this study, a carbon cementitious microfiltration membrane with high mechanical strength, small average pore size, good PWF, and porosity was prepared by adding PAC as an additive instead of silica powder into cementitious materials by dry pressing and curing at room temperature. CCM has the ability to catalyze ozone-based oxidation of a broad spectrum of organic compounds. The *k*_obs_ of the CCM–ozone coupling process was 1.6–4 times than that of ozone-based oxidation alone process. The doping of PAC did not reduce the catalytic oxidation efficiency of CM. CCM has a strong alkaline buffering effect and can produce a leveling effect on ozone-based oxidation to remove pollutants in the pH range of 4.0–10.0. The final pH values of the membrane alone system and the Ozone/CCM system were both around 9.6. The final pH of the Ozone/CCM + OR system was in the neutral range, while the pH of the membrane surface was 10.0, which was in the alkaline range. PAC in CCM can accelerate mass transfer, improve ozone decomposition efficiency, and increase ozone utilization in the reaction. CCM has the ability to catalyze the oxidation of organic compounds in a stable and continuous manner, which extends the application scope of CCM and provides a theoretical basis for the practical application of CCM.

## Figures and Tables

**Table 1:** Reaction kinetics of organic pollutants removal by three ozone oxidation systems.

Name	CM/Ozone		CCM/Ozone		Ozone Alone	
	*k*_obs_ (min^−1^)	R^2^	*k*_obs_ (min^−1^)	R^2^	*k*_obs_ (min^−1^)	R^2^
Nitrobenzene	0.1837	0.996	0.1934	0.995	0.0861	0.995
*p*-CA	0.7331	0.998	0.7907	0.998	0.9521	0.991
BP-4	0.2849	0.998	0.3932	0.994	0.0964	0.999
*p*-CP	0.6836	0.995	1.0053	0.999	0.2664	0.964
*p*-CNB	0.1406	0.996	0.1622	0.988	0.0834	0.978
*p*-CBA	0.2039	0.998	0.2117	0.997	0.1286	0.986

Conditions: pH = 6.9 ± 0.1, [O_3_] = 0.5 mg/L, [nitrobenzene]_0_ = [*p*-CA]_0_ = [BP-4]_0_ = [*p*-CP]_0_ = [*p*-CNB]_0_ = [*p*-CBA]_0_ = 0.064 mM.

## Data Availability

The data presented in this study are available on request from the corresponding author.

## Supplementary Materials

The following are available online at https://www.mdpi.com/article/10.3390/membranes11080601/s1, Text S1, Calculation of the rejection rate of SiO_2_ by the membrane; Text S2, Determination conditions of organic compounds; Table S1, Reaction kinetics of nitrobenzene by CCM-catalyzed ozonation with solutions through or not through the membranes in different initial pH conditions; Figure S1, Schematic diagram of pH value change measured in solution; Figure S2, Degradation of BP-4 by different ozonation systems in batch experiments; Figure S3, Reaction kinetics of organic pollutants by CM- and CCM-catalyzed ozonation; Figure S4, Normalized water permeance of the membrane after used.
