# Supplementary material for: Catalytic Efficiency of Carbon-Cementitious Microfiltration Membrane on the Ozonation-Based Oxidation of Small Molecule Organic Compounds and Its Alkaline Buffering Effect in Aqueous Solution
Source: Membranes (Basel). 2021 Aug 7;11(8):601. doi: 10.3390/membranes11080601 (PMC8399918; doi:10.3390/membranes11080601)
Supplement: Supplementary file 1 [file membranes-11-00601-s001.zip › membranes-1298803-supplementary.pdf]

Supporting information files for

Catalytic Efficiency of Carbon-Cementitious Microfiltration Membrane on the Ozonation-Based Oxidation of Small Molecule Organic Compounds and Its Alkaline Buffering Effect in Aqueous Solution

Jingyi Sun [1], Zhonglin Chen [1], Shan Liu [1], Jing Kang [1,*], Yuhao Guo [1], Liming Cai [1], Jimin Shen [1,*], Binyuan Wang [1], Shengxin Zhao [1] and Zilong Song [2]

[1] State Key Laboratory of Urban Water Resource and Environment, School of Environment, Harbin Institute of Technology, Harbin 150090, China; sunjingyihit@163.com (J.S.); zhonglinchen@hit.edu.cn (Z.C.); liumountainhit@163.com (S.L.); guoyuhaohit@163.com (Y.G.); cailiminghit@163.com (L.C.); wangbinyuan2008@163.com (B.W.); shengxin_zhao@163.com (S.Z.)

[2] Beijing Key Lab for Source Control Technology of Water Pollution, College of Environmental Science and Engineering, Beijing Forestry University, Beijing 100083, China; tiannonsongzilong@163.com

* Correspondence: jingkanghit@163.com (J.K.); shenjimin@hit.edu.cn (J.S.); Tel.: +86-0451-86283001

Content in this SI files

Text S1 Calculation of the rejection rate of SiO_2 by the membrane.

Text S2 Determination conditions of organic compounds.

Table S1 Reaction kinetics of nitrobenzene by CCM-catalyzed ozonation with solutions through or not through the membranes in different initial pH conditions.

Fig. S1 Schematic diagram of pH value change measured in solution.

Fig. S2 Degradation of BP-4 by different ozonation systems in batch experiments.

Fig. S3 Reaction kinetics of organic pollutants by CM- and CCM-catalyzed ozonation.

Fig. S4 Normalized water permeance of the membrane after used.

Text S1 Calculation of the rejection rate of SiO_2 by the membrane.

SiO_2 particle was used as the model particle to evaluate the separation of the membrane. The SiO_2 rejection rate was calculated as Eq. S(1):

$$R_{SiO_2} = \left(1-\frac{C_P}{C_0}\right)\times 100\% \qquad (1)$$

where R_{SiO2} is the rejection of SiO_2 (%), C_P is the turbidity of SiO_2 particle in effluent solution (NTU), C_0 is the turbidity of SiO_2 in influent solution (NTU). The initial turbidity of the SiO_2 particle is 20 NTU, and the trans-membrane pressure is 20 kPa.

Text S2 Determination conditions of organic compounds.

All of the organic models were determined by Ultra Performance Liquid Chromatography (UPLC, Agilent 1290 Infinity II, USA), and separated on the Agilent ZORBAX SB-C18 (4.6×150 mm, 5 μm) column. The column temperature was 30°C. The injection volume was 10 μL. The mobile phases of nitrobenzene, *p*-CNB and *p*-CA were all methanol: water of 70:30 (v/v). The detection wavelengths of UV detector were 262, 260 and 244 nm, respectively. The mobile phase of *p*-CP was acetonitrile and water with 50: 50 (v/v) ratio, and mobile phases of *p*-CBA and BP-4 were 0.1% formic acid in acetonitrile: water of 60:40 and 40:60 (v/v), respectively. The detection wavelengths of UV detector were 220, 236 and 285 nm, respectively. The isocratic elutions of organic compounds were performed at a flow rate of 1.0 mL min^{-1} except *p*-CBA was 0.8 mL min^{-1}.

Table S1 Reaction kinetics of nitrobenzene by CCM-catalyzed ozonation with solutions through or not through the membranes in different initial pH conditions.

pH_0	k_{obs} of not through-membrane (min^{-1})	R^2	k_{obs} of through-membrane (min^{-1})	R^2
4.0	0.0916	0.993	0.241	0.964
5.0	0.0964	0.997	0.227	0.971
6.0	0.105	0.978	0.199	0.994
7.0	0.109	0.976	0.234	0.983
8.0	0.114	0.981	0.223	0.972
9.0	0.117	0.965	0.237	0.968
10.0	0.094	0.996	0.214	0.950

Conditions: $[O_3]$ = 0.5 mg/L; $[nitrobenzene]_0$ = 0.064 mM.

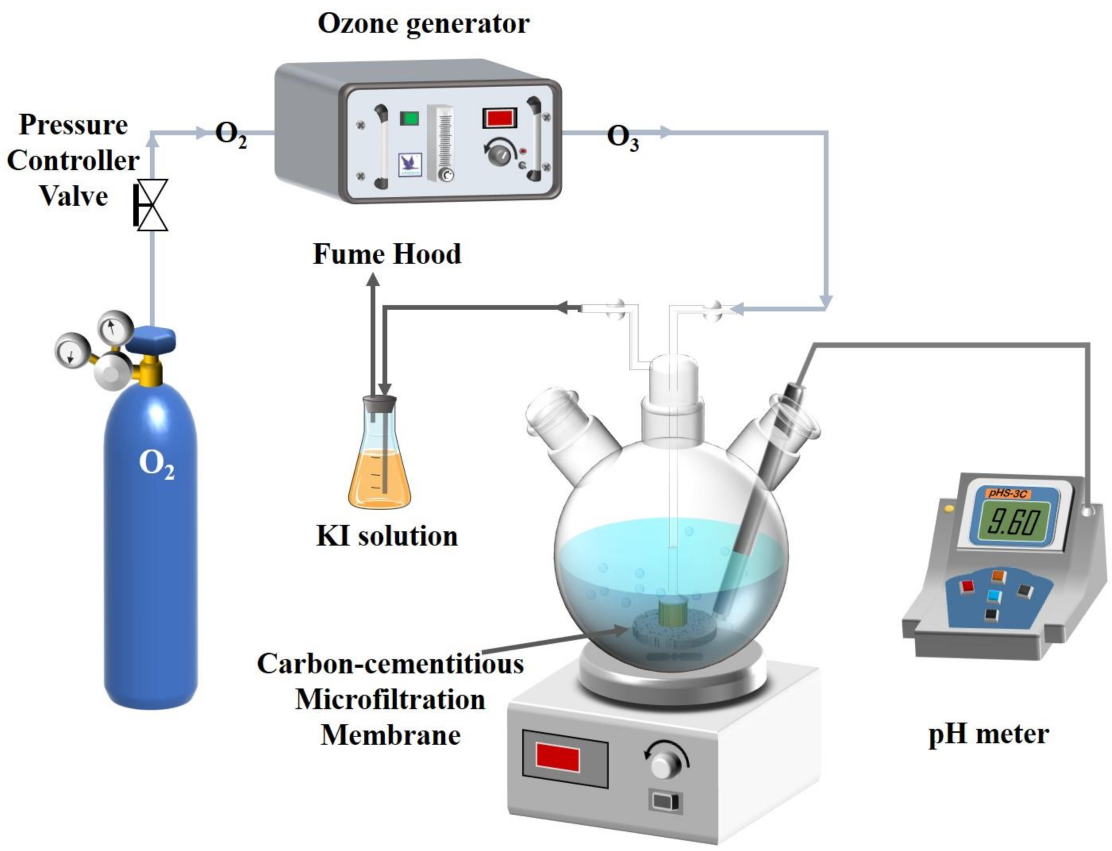

Fig. S1 Schematic diagram of pH value change measured in solution.

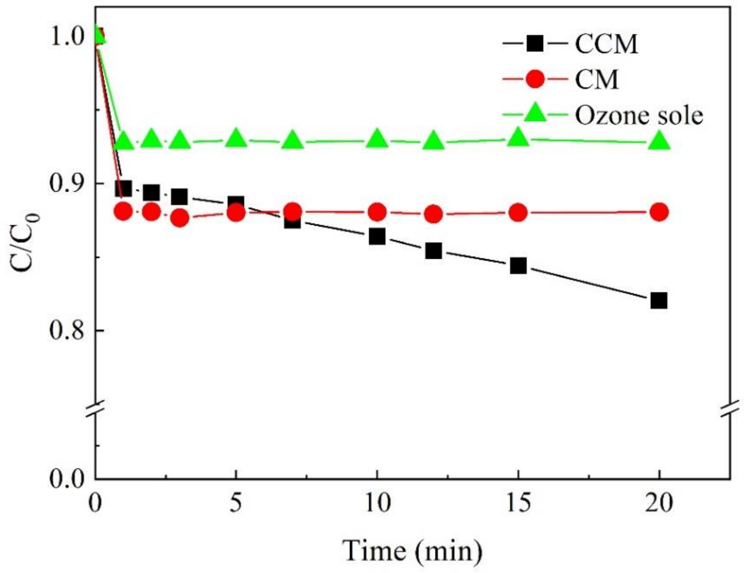

Fig. S2 Degradation of BP-4 by different ozonation systems in batch experiments.

Conditions: pH = 6.9±0.1, $[O_3]_0$ = 2 mg/L, $[BP\text{-}4]_0$ = 10 mg/L.

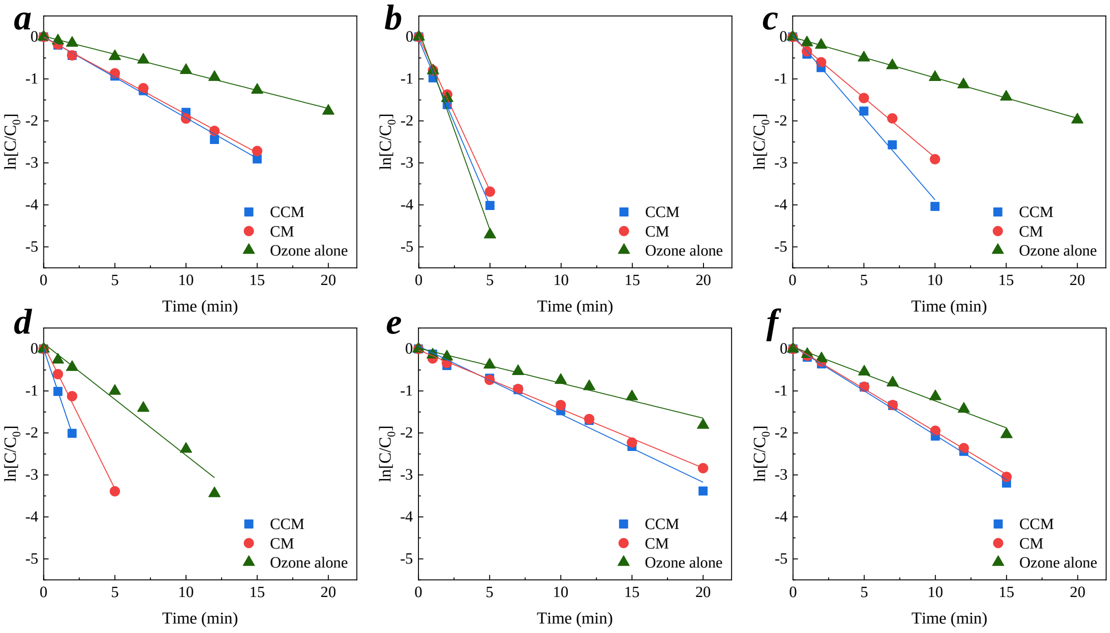

Fig. S3 Reaction kinetics of organic pollutants by CM- and CCM-catalyzed ozonation.

(a) nitrobenzene; (b) *p*-CA; (c) BP-4; (d) *p*-CP; (e) *p*-CNB; (f) *p*-CBA.

Conditions: pH = 6.9±0.1, $[O_3]$ = 0.5 mg/L; $[\text{nitrobenzene}]_0$ = 0.064 mM.

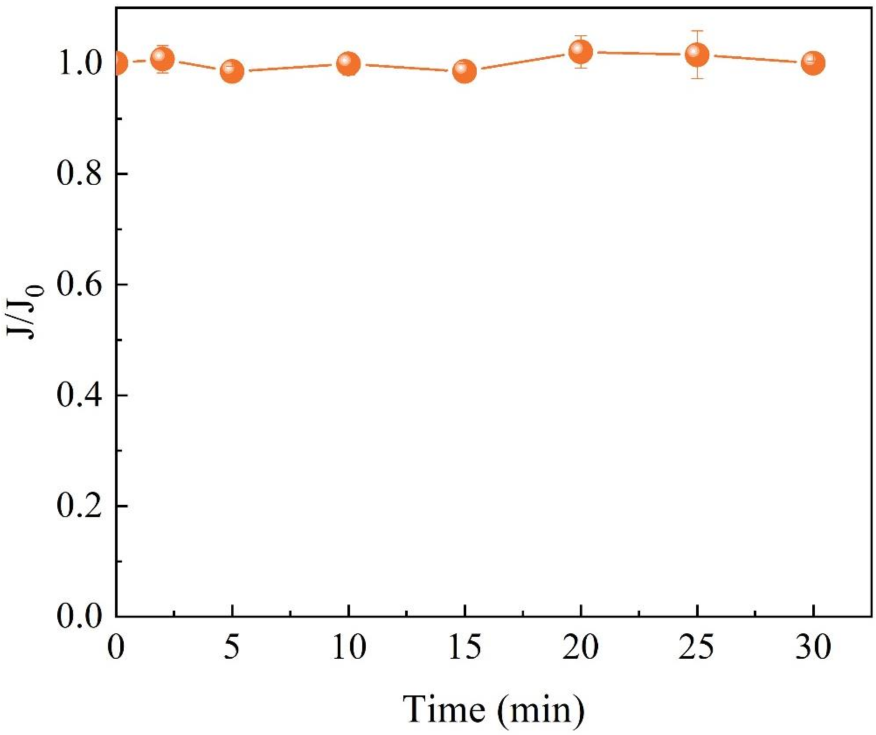

Fig. S4 Normalized water permeance of the membrane after used.
